# CT Radiomics for the Early Identification of Fungal Co-infection in Immunocompromised Patients with Viral Pneumonia

**DOI:** 10.2174/0115734056443124260427113549

**Published:** 2026-04-29

**Authors:** Le Zhou, Renjun Huang, Xinbing Zheng, Jie Xu, Qinghua Gu, Xiaoping Huang, Yonggang Li

**Affiliations:** 1 Department of Radiology, The First Affiliated Hospital of Soochow University, Suzhou City, Jiangsu Province 215000, China; 2 Department of Radiology, Zhangjiagang Sixth People's Hospital, Suzhou, China; 3 Center of Clinical Laboratory, the First Affiliated Hospital of Soochow University, Suzhou, China; 4 Department of Radiology, Suzhou Yongding Hospital, 1388 Gaoxin Road, Wujiang District, Suzhou, Jiangsu 215200, China; 5 Department of Infectious Diseases, The First Affiliated Hospital of Soochow University, Suzhou, China

**Keywords:** Computed tomography, Radiomics, Machine learning, Pulmonary infections, Viral pneumonia, Fungal co-infections, SARS-CoV-2, COVID-19

## Abstract

**Introduction:**

This study aimed to establish and validate CT-based radiomics models combined with clinical data to identify Fungal Co-Infections (FCI) in immunocompromised patients with Viral Pneumonia (VP).

**Materials and Methods:**

A total of 406 patients (VP: 283; FCI: 123) from two hospitals were retrospectively enrolled and divided into training (n = 218), testing (n = 96), and external validation (n = 92) cohorts. Radiomics features were extracted from chest CT images. Feature selection was performed using the Least Absolute Shrinkage And Selection Operator (LASSO), and logistic regression models were built with clinical, radiomics, and combined inputs. Model performance was assessed using the Area Under the Receiver Operating Characteristic Curve (AUC), calibration, and Decision Curve Analysis (DCA).

**Results:**

The combined model achieved AUCs of 0.981 (95% CI: 0.959 - 0.992), 0.845 (95% CI: 0.762 - 0.950), and 0.835 (95% CI: 0.715 - 0.937) in the training, testing, and external validation cohorts, respectively, and consistently outperformed clinical-only and radiomics-only models.

**Discussion:**

The model identified characteristic clinical and imaging differences between VP and FCI, including higher neutrophil counts, lower lymphocyte counts, and imaging markers such as reversed halo sign and solid nodules in FCI. These findings support the potential of radiomics as a noninvasive tool for early detection and risk stratification.

**Conclusion:**

CT-based radiomics provides an effective approach for differentiating VP and FCI in immunocompromised patients, with potential to improve diagnosis and clinical management.

## 1 INTRODUCTION

Viral Pneumonia (VP) remains a major global health challenge, particularly among immunocompromised individuals. Influenza viruses and severe acute respiratory syndrome coronavirus 2 (SARS-CoV-2), the causative agent of coronavirus disease 2019 (COVID-19), are among the most prevalent viral pathogens responsible for respiratory tract infections with substantial morbidity and mortality. Since the onset of the COVID-19 pandemic in late 2019, and in the context of seasonal and pandemic influenza outbreaks, clinicians have been faced with a growing number of critically ill patients suffering from severe viral pneumonia requiring hospitalization, intensive care, or mechanical ventilation [1-3].

Both COVID-19 and influenza-associated pneumonia lead to alveolar injury, diffuse lung inflammation, and impaired mucosal defenses. The frequent use of corticosteroids, immunomodulators, and broad-spectrum antibiotics further increases susceptibility. In patients with underlying immunosuppression, these factors increase the risk of secondary or co-infections, particularly invasive fungal infections such as pulmonary aspergillosis and candidiasis [4-8]. Once co-infection occurs, the patient's mortality rate and treatment difficulty increase significantly [9-11].

For patients suspected of pulmonary co-infection, antifungal drugs or corticosteroids are sometimes given as empirical therapy. However, inappropriate use can cause adverse effects and increase complications [7]. This highlights the need for timely and accurate diagnosis. A comprehensive approach may include histopathology, direct microscopy, microbial culture, (1,3)-β-D-glucan testing, and PCR-based assays. Each of these methods has important limitations. For example, microbial culture often takes a long time, depends on pathogen load, and can be influenced by operator bias [12]. Many laboratory tests also lack adequate specificity and sensitivity, leading to false-negative or false-positive results [13]. Invasive procedures such as lung biopsy are often not feasible in critically ill patients, especially those with coagulopathy or respiratory failure [12,14]. In addition, concerns over aerosol transmission have led many laboratories to suspend certain tests, such as direct microscopy and galactomannan assays of respiratory samples [6,8,15].

Radiological imaging, particularly chest Computed Tomography (CT), is commonly used to assess pulmonary infections. However, CT alone often fails to reliably differentiate Fungal Co-Infections (FCI) from viral pneumonia when characteristic radiological signs (*e.g.*, air crescent sign, reverse halo sign) are absent [12]. Furthermore, the presence of diffuse lung opacities (commonly referred to as “white lung”) in patients with severe pneumonia complicates interpretation, thereby reducing diagnostic accuracy and increasing the potential for misdiagnosis. These challenges underscore the urgent need for a more sensitive and specific diagnostic tool capable of accurately identifying co-infection.

Radiomics, an emerging field that extracts high-dimensional quantitative features from medical imaging, offers a promising avenue for capturing subtle differences in tissue texture, morphology, and intensity that may elude human observers. When integrated with machine learning algorithms, radiomic analysis has the potential to facilitate non-invasive, reproducible, and interpretable differentiation between viraland fungal pulmonary infections [16,17].

Several contributions have been made previously in lung infections using radiomics and deep learning approaches [18-20]. This study aims to develop and validate CT-based radiomics models to differentiate FCI from VP in immunocompromised patients.

## 2 MATERIALS AND METHODS

### 2.1 Study Population

This retrospective study was approved by the hospital's institutional review board (2022-292) with a waiver of informed consent, in compliance with the amended Declaration of Helsinki and applicable regulations governing retrospective data.

A total of 406 patients (mean age 70.1±11.4 years; 159 males and 247 females) were recruited from two independent centers. This sample size was considered sufficient to ensure model robustness and to allow both internal and external validation. Patients with a confirmed diagnosis of VP or FCI between October 2022 and May 2025 were included. VP in this study was caused exclusively by SARS-CoV-2 or influenza A/B.

VP was diagnosed based on laboratory confirmation of respiratory viral infection, defined as a positive Real-Time Polymerase Chain Reaction (RT-PCR) result for SARS-CoV-2 or influenza viruses obtained from nasopharyngeal swab specimens, in combination with compatible clinical symptoms and chest CT findings.

FCI was diagnosed based on microbiological evidence combined with compatible clinical and radiological findings. Major diagnostic tests included fungal culture of respiratory specimens, serum (1,3)-β-D-glucan assays, and galactomannan tests in serum or bronchoalveolar lavage fluid. All included cases met diagnostic criteria consistent with at least probable fungal infection.

Only immunocompromised patients, defined according to established clinical criteria [21], were eligible for enrollment. Immunocompromised status was determined based on the presence of at least one of the following conditions: (1) active non-pulmonary malignancy with recent chemotherapy or radiotherapy; (2) history of solid organ or stem cell transplantation requiring immunosuppressive therapy; (3) long-term use of immunosuppressive medications; (4) HIV infection with CD4+ count <200/μL; or (5) presence of chronic illnesses such as end-stage renal disease, liver cirrhosis, or poorly controlled diabetes. Patient eligibility was confirmed by two independent physicians through review of electronic medical records. Exclusion criteria: (1) Patients with malignant lung tumors; (2) Patients with significant image artifacts that prevent the drawing of a Volume of Interest (VOI). (3) Patients with a history of lung surgery. As patients underwent multiple chest CT scans to assess their condition after hospitalization, we selected the scan closest in time to the laboratory examination (the longest interval was 2 days, and the shortest was the same day).

### 2.2 CT Image Acquisition

All patients underwent non-contrast chest CT examinations using seven multi-detector scanners (SOMATOM Definition Flash, Siemens; Revolution CT, GE Healthcare; Aquilion, Toshiba; Brilliance 16, Philips; IQon Spectral CT, Philips; iCT 256, Philips; Discovery CT750 HD, GE Healthcare). The acquisition protocol was standardized across centers, with a tube voltage of 120 kVp, tube current-time product of 200-210 mAs, field of view (FOV) of 350 × 350 mm, and matrix size of 512 × 512. Images were initially acquired with a slice thickness of 5 mm and subsequently reconstructed into high-resolution thin sections (1.0-1.25 mm) using a lung kernel to ensure accurate depiction of pulmonary structures and pathological changes.

### 2.3 Radiological and Clinical Data

Clinical information was collected from electronic medical records (Table **1**). The chest CT images of all participants were exported from the Picture Archiving and Communication Systems (PACS) system workstations in the standardized Digital Imaging and Communications in Medicine (DICOM) format.

### 2.4 Image Segmentation

All CT images were processed using ITK-SNAP software (version 3.8.0, http://www.itksnap.org/). VOI encompassing the infected regions was delineated on lung-window images through a semi-automatic segmentation approach followed by manual adjustment. Segmentation was independently performed by two radiologists with 8 and 9 years of experience in thoracic imaging. To evaluate reproducibility, intra- and inter-observer agreement of radiomics features was assessed using the Intraclass Correlation Coefficient (ICC). A random subset of 50 patients (30 with VP and 20 with FCI) was re-segmented by Reader 2 after a four-week interval. The delineation process demonstrated good reproducibility, with intra-observer ICC of 0.82 (95% CI: 0.77-0.87) and inter-observer ICC of 0.80 (95% CI: 0.75-0.85). An illustrative schematic of the segmentation workflow is provided in Fig. (**1**).

### 2.5 Radiomics Feature Extraction

The extraction of radiomics features was conducted for each VOI utilizing the PyRadiomics package (Version 3.0.1, http://www.radiomics.io/pyradiomics.html), which was integrated into the Python platform (Version 3.7.1). The features were computed with a 1×1×1-mm^3^ resampled pixel spacing, and a bin width of 25 was utilized for extracting higher-order features. The extracted radiomics features encompassed four main categories: (1) texture features, (2) shape features, (3) first-order features, and (4) wavelet transform-based features. Subsequently, the extracted radiomics features underwent standardization using Z scores. The feature extraction process strictly adhered to the guidelines outlined by the Image Biomarker Standardization Initiative (IBSI) to ensure consistency and rigor in the analysis [22].

### 2.6 Feature Selection and Model Building

The clinical model, radiomics model, and combined model were constructed using Logistic Regression (LR) based on the selected features. Feature selection and model construction were initially conducted in the training cohort, followed by validation in the testing and external validation cohorts. The diagnostic performance of the models was evaluated using Receiver Operating Characteristic (ROC) curve analysis, with Area Under the Curve (AUC), and AUCs were compared using DeLong’s test. Calibration curves were generated to assess the calibration capability of the models, and Decision Curve Analysis (DCA) was applied to evaluate their clinical utility [23].

### 2.7 Statistical Analysis

Statistical analyses were performed using SPSS version 26.0 (IBM, USA) and R version 3.6.1 (http://www.r-project.org). Continuous variables with normal distribution are presented as mean ± standard deviation, whereas non-normally distributed variables are presented as median (interquartile range). Differences in continuous variables were compared using the Mann-Whitney U test or independent-samples t test, and differences in categorical variables were compared using the chi-squared test or Fisher’s exact test as appropriate. Highly correlated features (absolute Pearson correlation coefficient ≥ 0.9) were excluded to reduce redundancy. The LASSO algorithm was implemented using the “glmnet” package, and logistic regression models were trained in R. Statistical significance was set at *p* < 0.05.

## 3 RESULTS

### 3.1 Clinical and Radiological Characteristics

**Fig. (2):**
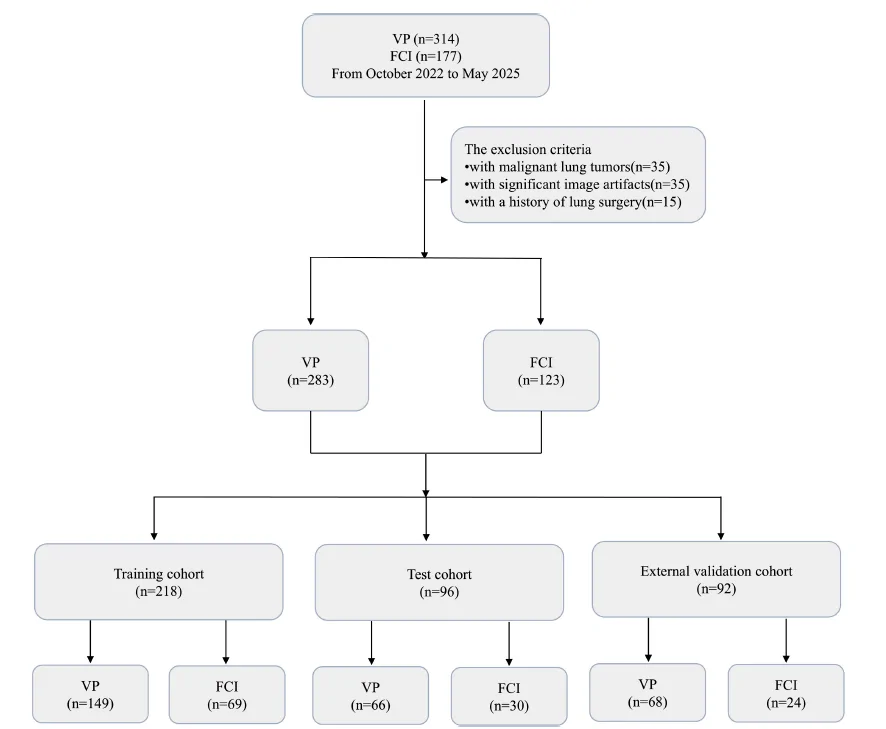
Flow diagram of patient selection.

In this study, a total of 406 patients were enrolled, including 283 patients with VP and 123 patients with FCI. Among them, 218 patients were assigned to the training cohort, 96 to the testing cohort, and 92 to the external validation cohort (Fig. **2**). The clinical and radiological characteristics of the study population are summarized in Table **1**. Among the 283 patients with VP, 178 (62.9%) were infected with SARS-CoV-2 and 105 (37.1%) with influenza A/B. The distribution of viral subtypes was comparable across the training, testing, and external validation cohorts. The positivity rates for major microbiological tests used to diagnose FCI are summarized in Table **S1**.

In the training, testing, and external validation cohorts, patients with FCI showed significantly higher neutrophil counts and percentages than those with VP (*p* < 0.01), whereas VP patients exhibited higher lymphocyte counts and percentages, and a greater prevalence of GGO (*p* < 0.05). In the testing cohort, FCI patients had higher leukocyte counts (*p* < 0.05) and a higher prevalence of the reversed halo sign (*p* < 0.01). Solid nodules were more frequent in FCI than VP patients in the testing and validation cohorts (*p* < 0.05), whereas atelectasis was more common in FCI across the training and testing cohorts (*p* < 0.05). In contrast, pleural effusion occurred more frequently in VP patients within the testing cohort. Other clinical and CT findings showed no statistically significant differences across the three cohorts.

### 3.2 Radiomics Features Selection and Model Development

**Fig. (3):**
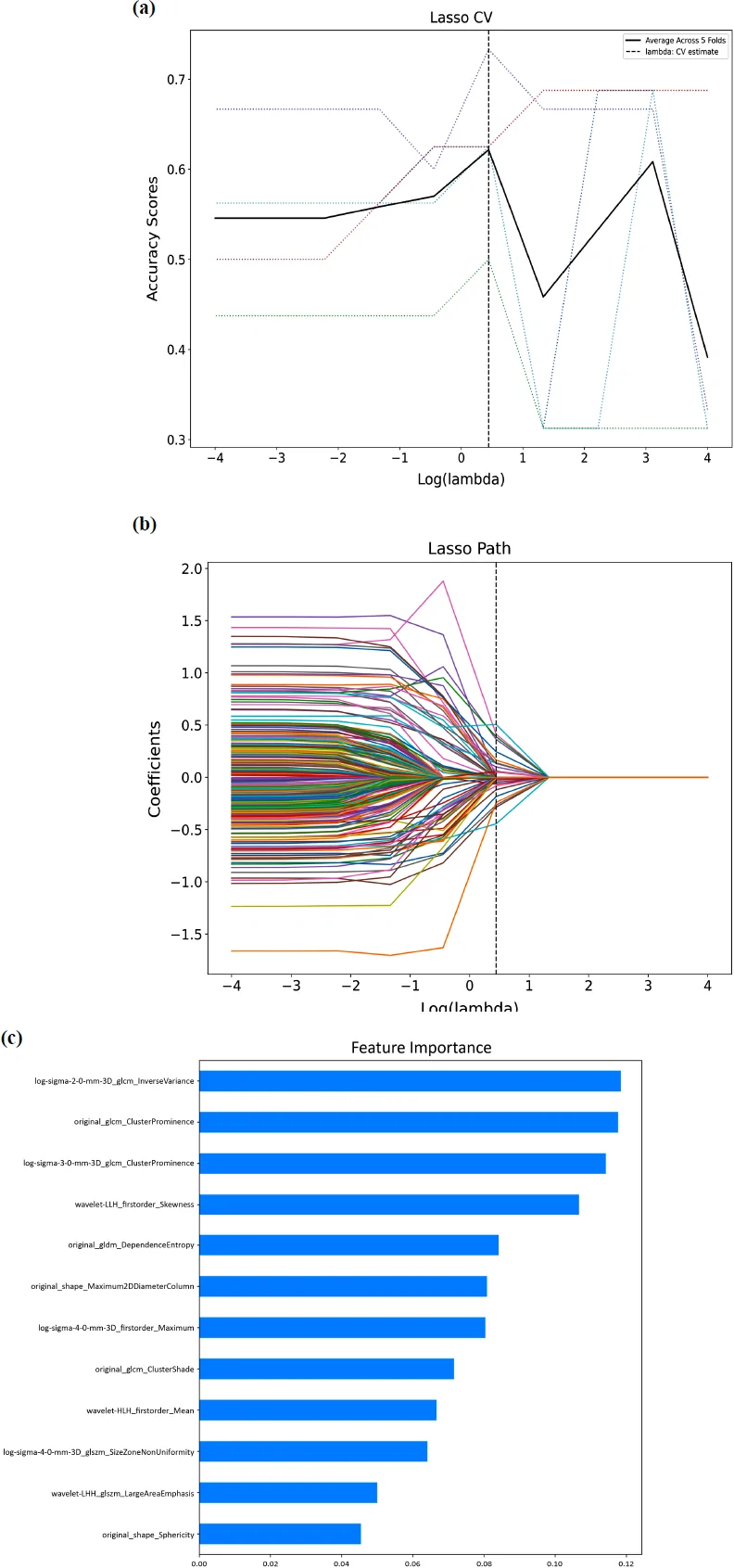
Feature selection for the radiomics model using the LASSO algorithm. (**a**) Five-fold cross-validation determined the optimal λ. (**b**) The vertical dotted line indicates feature coefficients at this λ. (**c**) Selected radiomics features and their corresponding coefficients are shown.

A total of 2688 radiomic features were systematically extracted from CT images. Redundant features were first reduced using Pearson correlation analysis, yielding 303 candidates. Subsequent selection with SelectPercentile and LASSO identified 12 discriminative features (Fig. **3**). The final combined model was developed by integrating these radiomic signatures with relevant clinical parameters.

### 3.3 Model Predictive Performance

**Fig. (5):**
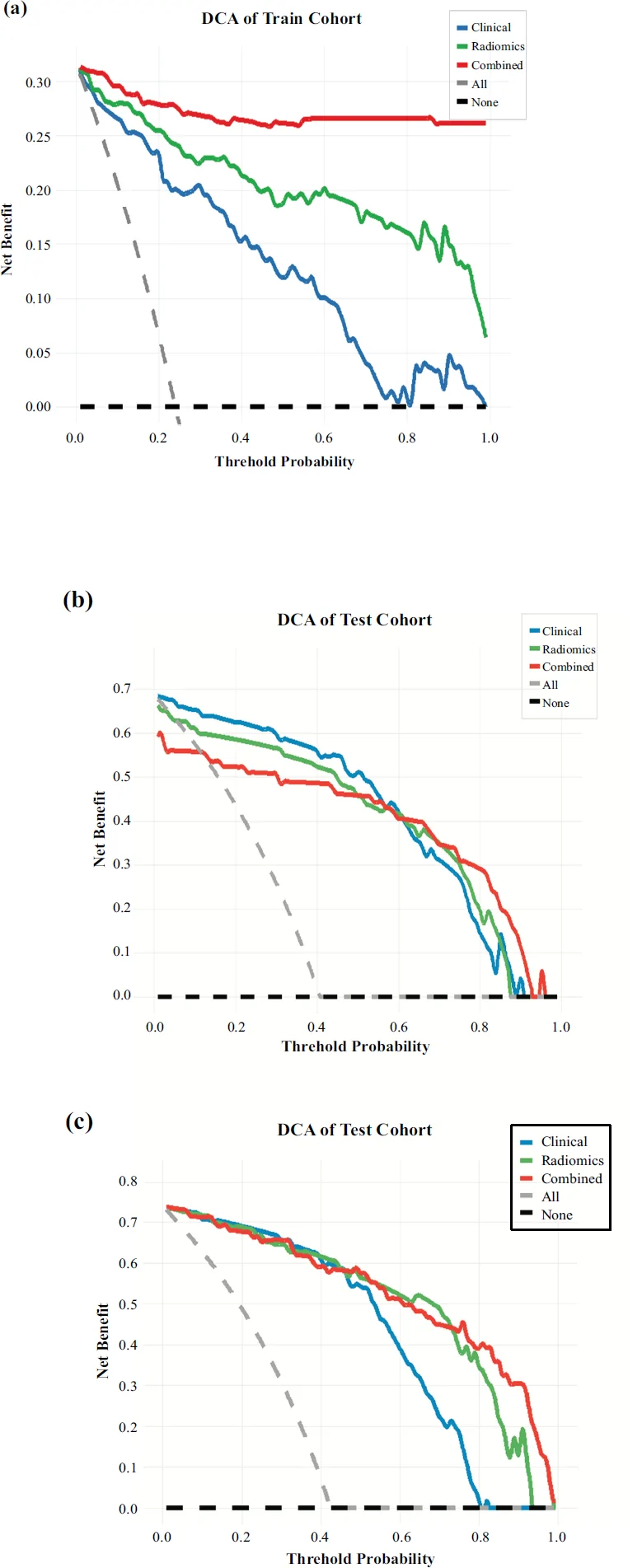
DCA of the clinical, radiomics, and combined models in the training (**a**), testing (**b**), and external validation cohorts (**c**).

**Fig. (6):**
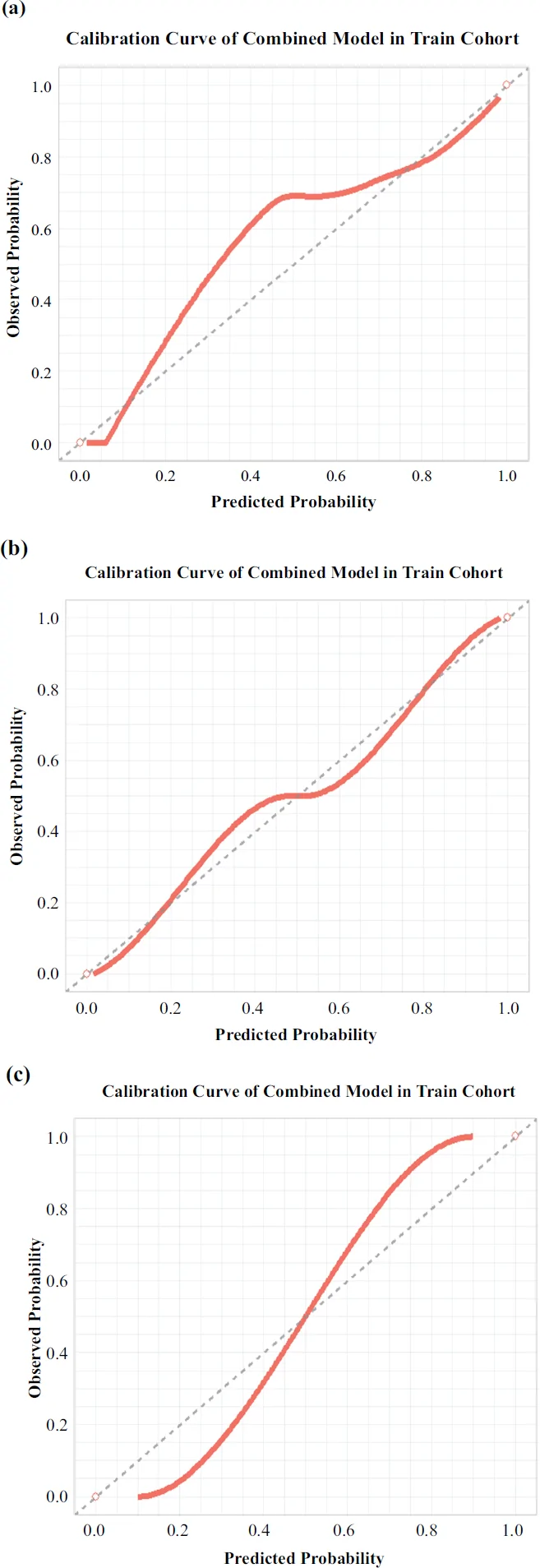
Calibration curves of the combined model in the training (**a**), testing (**b**), and external validation cohorts (**c**).

As shown in Fig. (**4**), the combined model consistently outperformed both the clinical and radiomics models across the training, test, and external validation cohorts, with AUCs of 0.981 (95% CI: 0.959 - 0.992), 0.845 (95% CI: 0.762 - 0.950), and 0.835 (95% CI: 0.715 - 0.937), respectively. DCA confirmed that all three models provided clinical net benefit (Fig. **5**). Moreover, the calibration curves (Fig. **6**) indicated excellent agreement between predicted and observed risks for the combined model across all cohorts.

## 4 DISCUSSION

In this study, CT-based radiomics models incorporating clinical features were developed and showed high accuracy in identifying fungal co-infection in immunocompromised patients with VP.

Although the combined model achieved a higher AUC in the training cohort, performance declined in the testing and external validation cohorts. This phenomenon is commonly reported in radiomics studies and may be attributed to differences in patient characteristics and imaging protocols across cohorts. Importantly, the model maintained stable performance in the external validation cohort, supporting its robustness and generalizability.

LR was selected as the primary modeling approach due to its robustness and interpretability, particularly in studies with moderate sample sizes and high-dimensional radiomics features. Compared with more complex machine-learning algorithms, LR provides greater transparency and a lower risk of overfitting, which is advantageous for clinical translation and decision support [24].

In the field of pneumonia diagnosis, radiomics and deep learning techniques have been increasingly utilized to address a variety of clinical challenges. These methods have been applied to distinguish VP from other types of pneumonia [18], to differentiate immune checkpoint inhibitor-related pneumonia in non-small cell lung cancer from radiation-induced pneumonia [20], and to classify mycoplasma pneumonia versus bacterial pneumonia [19]. Sun *et al.* developed a logistic regression-based predictive model to identify lung cancer patients at high risk of post-chemotherapy pulmonary infections, providing a practical tool for early surveillance and intervention [25]. Similarly, Shao *et al.* proposed a multimodal integration system incorporating clinical records, CT imaging, and laboratory findings, which enabled accurate diagnosis of pulmonary infections, pathogen identification, and prediction of severe disease progression [26]. Moreover, advanced imaging-based approaches have been employed to assess the severity of viral infections and estimate mortality risk in patients with pneumonia, particularly in intensive care unit settings [27].

In this study, the FCI group demonstrated higher neutrophil counts and percentages, consistent with intensified inflammatory responses and immune dysregulation in fungal co-infections [28]. Conversely, lymphocyte counts and percentages were significantly lower in the FCI group, likely due to combined immune suppression in VP with secondary fungal infection and direct impairment of lymphocyte function and survival by fungal pathogens [29,30]. Regarding imaging findings, GGO was more prevalent in the VP group across all cohorts, reflecting its well-recognized role as a hallmark of viral pneumonia, including COVID-19 [31].

In the testing cohort, leukocyte counts and the reversed halo sign were higher in the FCI group, the former reflecting stronger systemic inflammation [32], and the latter serving as a relatively specific radiological marker of pulmonary fungal infections such as invasive aspergillosis and mucormycosis [33]. In contrast, pleural effusion was more common in VP, probably due to diffuse inflammation and increased vascular permeability, whereas fungal infections tend to cause localized parenchymal lesions with limited pleural involvement [26].

Furthermore, in the testing and external validation cohorts, solid nodules were more frequent in the FCI group, consistent with angioinvasive fungal growth leading to necrosis and localized consolidation, whereas viral pneumonia usually manifests as diffuse GGO or patchy infiltrates [34].

Finally, atelectasis was more common in the FCI group across the training and testing cohorts, likely secondary to airway obstruction from fungal invasion and mucus plugging, consistent with prior studies in immunocompromised hosts [35].

DCA was performed to assess the potential clinical utility of the proposed model. Importantly, the model is not intended to directly dictate the initiation of antifungal treatment. Instead, it may assist clinicians in risk stratification by identifying patients with viral pneumonia who are at increased risk of fungal co-infection. In practice, such information could prompt closer clinical surveillance, earlier microbiological investigations, or multidisciplinary evaluation, thereby supporting timely and individualized management decisions.

The clinical variables included in the model were selected based on their biological relevance to fungal infection risk and systemic inflammatory response, as well as their accessibility in routine clinical practice. However, this study may not have captured all potential confounding factors. Variables such as the timing and intensity of immunosuppressive therapy, prior antifungal exposure, and other host-related factors were not fully accounted for and may have influenced model performance. Despite these limitations, the model maintained stable performance in the external validation cohort, suggesting a certain degree of robustness.

In this study, lesion segmentation was performed using a semi-automated approach, followed by manual refinement to ensure accurate delineation of heterogeneous pulmonary infections. While this strategy is feasible and commonly adopted in radiomics research, it may limit efficiency in routine clinical practice. Importantly, the radiomics pipeline developed in this study is compatible with emerging automated and semi-automated deep learning-based segmentation techniques, which have shown promising performance in lung and lesion segmentation. Integration of such methods may substantially improve efficiency and facilitate clinical translation in future applications.

To our knowledge, this is the first study to apply CT-based radiomics for evaluating fungal co-infections in immunocompromised patients with VP. The model provides a non-invasive method for risk stratification and may allow earlier detection when imaging features overlap. Integrated into radiology workflows, it could help standardize image interpretation and guide patient management. In summary, CT-based radiomics shows promise as a valuable tool in this setting and may assist in guiding clinical treatment and improving patient care.

## 5 STUDY LIMITATIONS

This study has several limitations. First, its retrospective design may have introduced selection bias. Second, although data were collected from two centers, the sample size was relatively limited, and heterogeneity in CT scanners and acquisition protocols across centers may have influenced radiomics features. Third, we focused on detecting fungal co-infections without differentiating specific fungal strains. Future studies with larger, multicenter cohorts and standardized imaging protocols are warranted to further enhance generalizability and robustness.

## CONCLUSION

This study established and validated CT-based radiomics models to distinguish VP from FCI in immunocompromised patients. The models offer a noninvasive tool that may support earlier recognition of FCI, improve diagnostic confidence, and inform timely treatment decisions. These findings highlight the potential of radiomics to complement clinical assessment and imaging interpretation, contributing to more precise management of high-risk patients.

## Figures and Tables

**Fig. (1):**
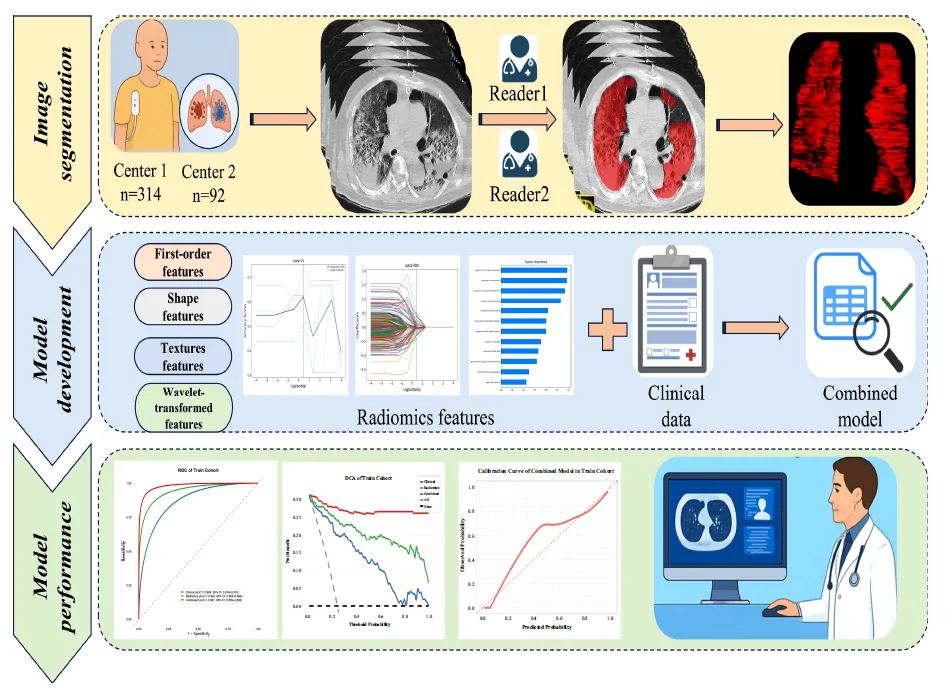
Flowchart of this study.

**Fig. (4):**
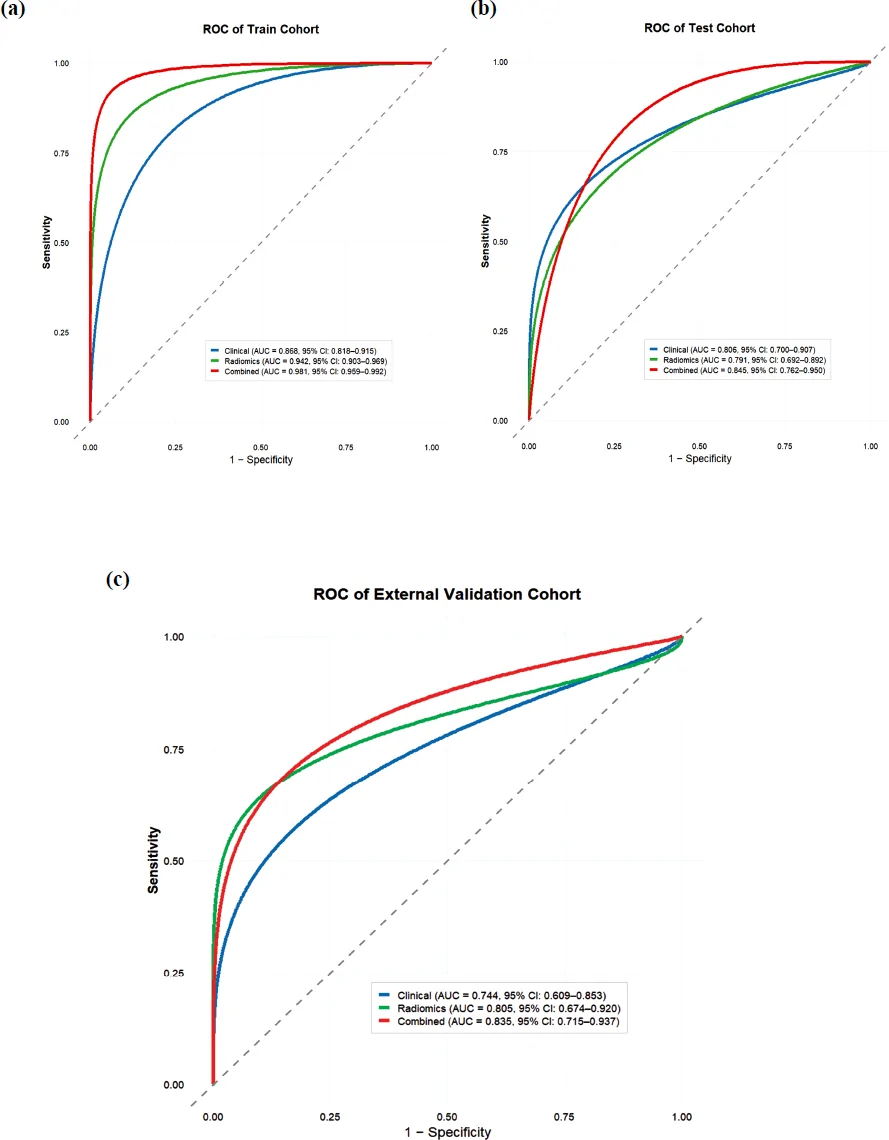
Receiver Operating Characteristic (ROC) curves of the clinical, radiomics, and combined models in the (**a**) training, (**b**) testing, and (**c**) external validation cohorts.

**Table 1:** Baseline clinical and radiological characteristics of patients in different cohorts.

**Characteristics**	**Training cohort (n=218)**			**Testing cohort (n=96)**			**External validation (n=92)**			*p*-value
	**VP**	**FCI**	***p*-value**	**VP**	**FCI**	***p*-value**	**VP**	**FCI**	*p*-value	-
	**(n=149)**	**(n=69)**	-	**(n=66)**	**(n=30)**	-	**(n=68)**	**(n=24)**	-	-
Age(years)	70.41±11.03	71.48±14.12	0.579	69.75±10.14	70.55±10.58	0.729	68.45±10.44	69.15±11.14	0.790	0.317
Neutrophil count (×10^9^/L)	3.93(2.35,7.22)	7.36(3.37,10.81)	<0.001***	4.06(3.00,5.93)	6.32(4.30,9.37)	<0.001***	3.96(2.38,7.26)	6.41(4.30,9.37)	0.001**	0.002**
Neutrophil percentage (%)	73.15(63.48,83.90)	85.10(81.60,91.20)	<0.001***	74.60(64.08,84.23)	84.60(68.20,92.00)	0.003**	73.60(63.18,83.03)	84.10(80.40,90.28)	<0.001***	0.005**
Monocyte count (×10^9^/L)	0.38(0.26,0.66)	0.36(0.28,0.63)	0.620	0.36(0.22,0.61)	0.37(0.22,0.58)	0.680	0.37(0.24,0.60)	0.36(0.23,0.59)	0.764	0.317
Monocyte percentage (%)	7.30(3.85,9.88)	6.40(3.35,8.75)	0.230	7.55(5.08,10.08)	6.50(3.50,8.10)	0.072	7.60(4.95,9.98)	6.42(3.49,8.50)	0.134	0.406
Lymphocyte count (×10^9^/L)	0.87(0.57,1.34)	0.57(0.33,0.77)	<0.001***	0.86(0.49,1.02)	0.65(0.34,0.79)	0.007**	0.85(0.53,1.12)	0.67(0.43,0.97)	0.046*	0.014*
Lymphocyte percentage (%)	17.15(10.78,26.75)	9.10(4.05,10.45)	<0.001***	18.43(11.44,28.72)	9.24(4.15,10.66)	<0.001***	15.15(9.54,24.21)	9.23(5.10,11.05)	<0.001***	0.004**
Leukocyte count (×10^9^/L)	5.36(3.74,9.29)	6.10(3.95,12.44)	0.320	5.48(3.72,10.11)	9.28(4.63,11.88)	0.011*	5.59(4.16,10.22)	6.01(3.65,10.21)	0.484	0.033*
Gender	-	-	0.805	-	-	0.515	-	-	0.408	0.093
Male	58(38.9%)	28(40.6%)	-	22(33.3%)	8(26.7%)	-	30(44.1%)	13(54.2%)	-	-
Female	91(61.1%)	41(59.4%)	-	44(66.7%)	22(73.3%)	-	38(55.9%)	11(45.8%)	-	-
Solid nodules	-	-	0.436	-	-	0.031*	-	-	0.028*	0.805
Negative	119(79.9%)	52(75.4%)	-	55(83.3%)	19(63.3%)	-	55(80.9%)	14(58.3%)	-	-
Positive	30(20.1%)	17(24.6%)	-	11(16.7%)	11(36.7%)	-	13(19.1%)	10(41.7%)	-	-
Reversed halo sign	-	-	0.676	-	-	0.001**	-	-	0.482	<0.001***
Negative	146(98.0%)	67(97.1%)	-	66(100%)	25(83.3%)	-	58(85.3%)	19(79.2%)	-	-
Positive	3(2.0%)	2(2.9%)	-	0(0%)	5(16.7%)	-	10(14.7%)	5(20.8%)	-	-
Atelectasis	-	-	0.024*	-	-	<0.001***	-	-	0.983	0.109
Negative	127(85.2%)	50(72.5%)	-	61(92.4%)	16(53.3%)	-	48(70.6%)	17(70.8%)	-	-
Positive	22(14.8%)	19(27.5%)	-	5(7.6%)	14(46.7%)	-	20(29.4%)	7(29.2%)	-	-
GGO	-	-	0.038*	-	-	0.001**	-	-	<0.001***	<0.001***
Negative	3(2.0%)	6(8.7%)	-	0(0%)	5(16.7%)	-	6(8.8%)	11(45.8%)	-	-
Positive	146(98.0%)	63(91.3%)	-	66(100%)	25(83.3%)	-	62(91.2%)	13(54.2%)	-	-
Pleural effusion	-	-	0.261	-	-	<0.001***	-	-	0.964	0.083
Negative	36(24.2%)	12(17.4%)	-	8(12.1%)	14(46.7%)	-	23(33.8%)	8(33.3%)	-	-
Positive	113(75.8%)	57(82.6%)	-	58(87.9%)	16(53.3%)	-	45(66.2%)	16(66.7%)	-	-

**Abbreviation:** GGO: Ground glass opacity;

**Note:** *p < 0.05; **p < 0.01; ***p < 0.001.

## Data Availability

The data and materials used in this study are available upon reasonable request by the corresponding author [Y.L.].

## LIST OF ABBREVIATIONS

FCI: Fungal Co-Infections
DCA: Decision Curve Analysis
LASSO: Least Absolute Shrinkage And Selection Operator
VP: Viral Pneumonia
CT: Computed Tomography
DICOM: Digital Imaging and Communications in Medicine

## STANDARDS OF REPORTING

STROBE guidelines were followed.
