# Supplementary material for: CT Radiomics for the Early Identification of Fungal Co-infection in Immunocompromised Patients with Viral Pneumonia
Source: Curr Med Imaging. 2026 Apr 29;22:E15734056443124. doi: 10.2174/0115734056443124260427113549 (PMC13613306; doi:10.2174/0115734056443124260427113549)
Supplement: Supplementary file 1 [file CMIM-22-E15734056443124_SD1.pdf]

1875-6603/26 *Send Orders for Reprints to* reprints@benthamscience.net

BENTHAM SCIENCE

CrossMark

Current Medical Imaging

Content list available at: https://benthamscience.com/journals/cmir

Supplementary Material

CT Radiomics for the Early Identification of Fungal Co-infection in Immunocompromised Patients with Viral Pneumonia

Le Zhou[1,#], Renjun Huang[1,#], Xinbing Zheng[2,*], Jie Xu[3], Qinghua Gu[4,*], Xiaoping Huang[5] and Yonggang Li[1,*]

[1]*Department of Radiology, The First Affiliated Hospital of Soochow University, Suzhou City, Jiangsu Province 215000, China*
[2]*Department of Radiology, Zhangjiagang Sixth People's Hospital, Suzhou, China*
[3]*Center of Clinical Laboratory, the First Affiliated Hospital of Soochow University, Suzhou, China*
[4]*Department of Radiology, Suzhou Yongding Hospital, 1388 Gaoxin Road, Wujiang District, Suzhou, Jiangsu 215200, China*
[5]*Department of Infectious Diseases, The First Affiliated Hospital of Soochow University, Suzhou, China*

Table S1. Positivity rates of major microbiological tests for FCI.

Diagnostic Test	No. tested (FCI)	No. positive	Positivity rate (%)
Fungal culture (respiratory specimens)	47	14	29.8
Serum (1,3)-β-D-glucan	42	25	59.5
Galactomannan (serum or BALF)	34	15	44.1

© 2026 The Author(s). Published by Bentham Science Publisher.

This is an open access article distributed under the terms of the Creative Commons Attribution 4.0 International Public License (CC-BY 4.0), a copy of which is available at: https://creativecommons.org/licenses/by/4.0/legalcode. This license permits unrestricted use, distribution, and reproduction in any medium, provided the original author and source are credited.

HOW TO CITE:
Zhou L, Huang R, Zheng X, Xu J, Gu Q, Huang X, Li Y. CT Radiomics for the Early Identification of Fungal Co-infection in Immunocompromised Patients with Viral Pneumonia. Curr Med Imaging, 2026; 22: e15734056443124. http://dx.doi.org/10.2174/0115734056443124260427113549
